# Le mal de Pott: à propos de 82 cas

**DOI:** 10.4314/pamj.v8i1.71078

**Published:** 2011-03-13

**Authors:** Badr Fedoul, Khalid Chakour, Mohamed El Faiz Chaoui

**Affiliations:** 1 Service de neurochirurgie, CHU Hassan II, Fès, Maroc

**Keywords:** Mal de pott, spondylodiscite, tuberculose, vertébrale

## Abstract

Nous rapportant dans cette étude, les résultats de l'expérience du service de neurochirurgie du CHU Hassan II de Fès dans la prise en charge du mal de pott dans la région de Fès. Il s'agit d'une étude rétrospective de quatre-vingt-deux cas; étalée sur une période de cinq ans (janvier 2002 au décembre 2006). L'objectif de ce travail était d'illustrer les différents aspects épidémiologiques, diagnostiques et thérapeutiques de la localisation vertébrale de la tuberculose dans notre pratique. L'âge moyen de nos patients était de 43,1 ans, avec une légère prédominance féminine (53,82%). La durée d'évolution de la maladie était longue (dix mois en moyenne); ceci est expliquée par la symptomatologie initiale insidieuse faite de rachialgies (98,78%) et une admission des patients au stade de complications neurologiques (41,46%). La radiographie standard était réalisée chez tous nos patients, et complétée par la TDM dans 86.58% des cas ce qui a permis de déceler la prédominance de l'atteinte dorsale et lombaire. L'IRM est l'examen de choix, elle était demandée chez tous les malades déficitaires (37,8%).Tous nos patients ont bénéficié d'un traitement antibacillaire associé à une immobilisation du foyer pottique. Une décompression par voie antérieure était réalisée chez 29 patients (35,36%); alors que la laminectomie n'était pratiquée que chez 5 patients (6.09%), tandis que l'évacuation de l'abcès de psoas était réalisée chez 25 patients (30,48%). Le diagnostic de certitude histologique était posé dans 51 cas (62,19%). Les meilleurs résultats étaient obtenus chez les malades opérés par voie antérieure, 26 cas (89,65%) de récupération totale et 3 cas (10,34%) partielle. L'évolution vers la consolidation et la fusion vertébrale était la règle chez tous nos malades et ceci au bout de 4 à 18 mois après le traitement.

## Introduction

Le mal de Pott correspond à la localisation du processus infectieux tuberculeux (dû au *Mycobacterium tuberculosis*) sur un ou plusieurs ensembles disco-vertébraux. La spondylodiscite tuberculeuse qui réalise la forme classique du mal du Pott est caractérisée par l'atteinte du disque intervertébral (DIV) et des deux vertèbres adjacentes. C'est une forme grave par l'atteinte neurologique qui peut être importante et définitive, mettant en jeu le pronostic fonctionnel. Les rachis dorsal et lombaire sont les plus fréquemment atteints dans 80% des cas [1,2]. La destruction vertébrale due à la tuberculose peut entraîner des déformations rachidiennes réalisant une cyphose ou gibbosité pottique. Le mal de Pott peut avoir des expressions cliniques variables, allant de la simple douleur rachidienne, aux formes graves qui peuvent associer des troubles neurologiques sévères et des déformations rachidiennes importantes. L'objectif de cette étude était d'illustrer les différents aspects épidémiologiques, diagnostiques et thérapeutiques de la localisation vertébrale de la tuberculose chez un groupe de patients admis au service de neurochirurgie du CHU Hassan II des Fès de Janvier 2002 à Décembre 2006. 

## Méthodes

Il s'agit d'une étude rétrospective de 82 patients, atteints de tuberculose vertébrale (mal de Pott) colligés au service de neurochirurgie du CHU Hassan II de Fès de Janvier 2002 à Décembre 2006. Le diagnostic de mal de Pott était retenu sur des preuves histologiques, mais aussi sur des arguments cliniques, biologiques et surtout radiologiques (TDM et IRM). L'analyse descriptive des variables cliniques et démographiques des patients a été présentée sous forme de pourcentage.

## Résultats

Sur le plan épidémiologique, le nombre de cas par an était estimé à 20. L'âge moyen de nos patients était de 43,1 ans (allant de 3 ans et demi à 73 ans), avec une légère prédominance féminine (53.8%). Plus de 60% de nos patients étaient d'origine urbaine. L'existence d'un contage tuberculeux récent était rapporté chez 17 cas (20.7%), alors que 14 de nos patients (17%) avaient un antécédent de tuberculose et 8 patients (9.7%) ont présenté une atteinte vertébrale, alors qu'ils étaient sous traitement antibacillaire pour une tuberculose extra rachidienne. 

Le délai de consultation entre les premiers symptômes et la confirmation du diagnostic était en moyenne de 10 mois. Sur le plan clinique 98.7% des patients ont consulté pour des douleurs rachidiennes associées à une altération de l'état général, un amaigrissement et une anorexie. La raideur rachidienne était retrouvée chez 96.3% des cas associée à des déformations rachidiennes à type de cyphose ou de scoliose dans 34.1% des cas. Le déficit neurologique était noté chez 41.4% des cas, avec une paraparésie chez 39% des cas, répartit selon le grading de Frankel en 2 cas grade A (2.43%) ; 14 cas grade B (17.07%) ; 12 cas grade C (14.63%) et 4 cas grade D (4.87%). Nous n'avons enregistrés que 2 cas (2.4%) de tétraparésie dont 1 grade C et un grade D. 

Tous nos patients ont bénéficié d'une radiographie standard complétée par une tomodensitométrie (TDM) dans 86.5% des cas. L'IRM médullaire était demandée d'emblée chez presque tous les malades présentant un déficit. La recherche d'une atteinte pulmonaire associée était systématiquement recherchée par la radiographie de poumon face. 

**Figure 1:**
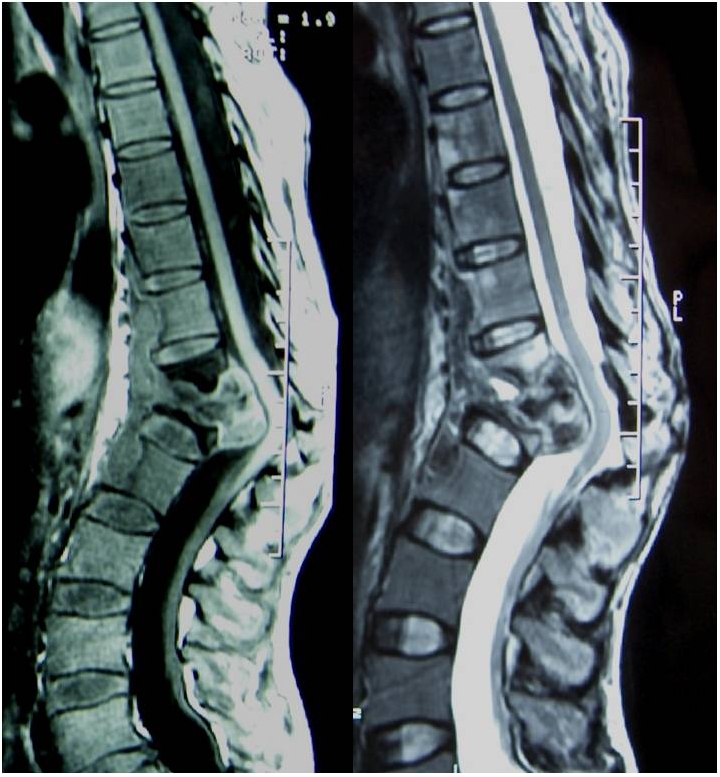
IRM dorso-lombaire en coupe sagittale T1 avec injection de Gadolinium+ séquence T2 chez un patient avec mal de Pott, montrant une spondylodiscite au niveau D12-L1 avec un énorme abcès intracanalaire et une déformation en cyphose et des signes de souffrance médullaire en regard

Nous avons noté une nette prédominance de l'atteinte dorsale et lombaire, répartie en 52.4% pour le siège lombaire et 41.4% pour le rachis dorsal (Figure 1). L'intradermoréaction à la tuberculine était réalisée chez 85.3% et était positive chez 36.5% des cas. Sur 30 prélèvements effectués on a pu isoler le BK que dans seulement 4 prélèvements. Nous avons adopté un traitement médical associé à une immobilisation rachidienne dans 28% des cas, alors que 71.9% ont bénéficié d'un traitement chirurgical associé à un traitement médical. 

**Figure 2:**
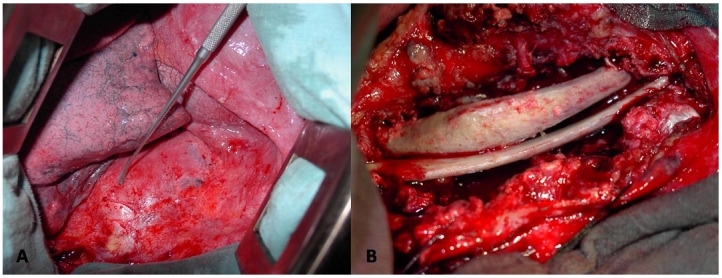
Mal de Pott; (A): une thoracotomie droite, après l'ouverture de l'espace inter-costal, les côtes sont écartées par un écarteur Finoquietto,on tombe sur un abcès paravertébral bombant. (B): Après la résection des séquestre osseux et la décompression canalaire et la réalisation d'une gouttière étendue du niveau sus au niveau sous-jacent à la lésion, un greffon iliaque et/ou costal (sans ostéosynthèse) est encastré après la réduction d'éventuelle cyphose par compression postérieure

Pour l'accès direct du foyer pottique, l'abord antérieur nous a permis l'évacuation de l'épidurite, la décompression canalaire, et la réduction parfaite des déformations rachidiennes, après une arthrodèse inter-somatique par un greffon iliaque et/ou costal et sans ostéosynthèse. Cet abord était réalisé chez 35.3% de nos patients. Au niveau du rachis dorsal une thoracotomie droite était réalisée chez 21 cas (Figure 2). Au niveau de la charnière dorso-lombaire D11-L2 une thoracophrénolombotomie nous a permis un abord large mettant en communication la cavité pleurale avec le rétro-péritoine après section du diaphragme. Cet abord était pratiqué chez 7.3% des cas. Au niveau du rachis lombaire, la voie d'abord postérieur consistant à une laminectomie surtout chez des patients présentant une compression plus postérieure n'était réalisé que chez 6% des cas; la lombotomie avec mise à plat et évacuation d'un abcès du psoas était pratiqué chez 30.4% des cas. Soixante et un prélèvements biopsiques étaient réalisés, l'étude anatomopathologique avait mis en évidence un granulome avec nécrose caséeuse chez 51 patients, alors que dans 10 cas, elle était non spécifique. Tous nos patients avaient bénéficié d'une immobilisation rachidienne pendant 3 mois jusqu'à la consolidation radiologique du foyer pottique. La rééducation était un complément thérapeutique indispensable chez tous les patients déficitaires. L'évolution chez les malades non opérés était favorable. Chez les malades opérés par voie antérieure, nous avons enregistré chez tous nos patients une récupération spectaculaire du déficit neurologique, qui était totale dans 89.6% des cas, et partielle dans 10.3% des cas. Sur les 5 patients opérés par voie postérieure; l'amélioration du déficit neurologique n'était enregistré que chez un patient. A noter que 4 patients opérés par voie postérieure ont présentés une aggravation de leur cyphose après la réalisation de la laminectomie. 

Au cours de notre étude, nous avons enregistré des complications liées au décubitus chez 17% des patients; avec 4,8% d'escarres sacrés et trochantériennes; 6% d'infection urinaire et 3,6% de phlébite. Nous avons déplorés 3 décès secondaires aux décompensations de tares. L'évolution radiologique des lésions tuberculeuses était marquée par la consolidation du foyer lésionnel et la formation du bloc vertébral dans un délai variables allant de 4 à 18 mois. 

## Discussion

L'histoire du mal de Pott a commencé avec Percival Pott entre 1779 et 1783, décrivant une impotence des membres inférieurs suite à une courbure de l'épine. Ce mal caractérisé par des abcès, une gibbosité et des paralysies portera son nom Â mal vertébrale de Pott Â [3]. 

L'emplacement géographique et la période de recrutement sont deux paramètres qui conditionnent l'incidence de la tuberculose. Le premier explique l'incidence élevée dans les pays en voie de développement par rapport aux pays développés. Pour le second, Flipo a montré que l'incidence annuelle du mal de pott était stable entre 1966 et 1991 pour ré-augmenter à 4 cas en 1992; 5 en 1993 et 1994. Cette augmentation d'incidence était liée à la période d'immigration des nord africains et l'augmentation du taux des sujets HIV positif [4]. Dans notre série, et puisque le Maroc est un pays d'endémie tuberculeuse, nous avons enregistré une incidence stable de 20 cas par an. 

Dans la série de Maftah et al [5] 54% des patients était de sexe féminin, ce qui rejoint nos résultats; alors que la série de Ghadouane et al [6] a montré une nette prédominance masculine (79.31%), ceci est expliqué par la population étudiée par Ghadouane et al. Pour Loembe et al [7] il y a une égalité de fréquence dans le sexe. L'existence de lésion tuberculeuse évolutive ou cicatricielle constitue un bon argument en faveur de la nature tuberculeuse des lésions. Cette éventualité était retrouvée dans 24.3% des cas par Maftah et al [5], ce chiffre avoisine celui de notre série. La notion de contage tuberculeux dans la série de Maftah et al était de 16.5% [5], et chez Ghadouane et al dans 20.6% des cas, ce qui concorde avec nos résultats. Le délai de diagnostic est généralement long entre 5 et 12 mois dans les séries suscitées [5-7]. Ce délai long est expliqué par la formation d'abcès intramusculaire constituant une forme de drainage du foyer tuberculeux après sa mise en tension; ce qui permet une meilleure tolérance de la symptomatologie et donc un retard diagnostique. 

Les douleurs rachidiennes constituent le symptôme majeur de la maladie qui amène le patient à consulter dans presque 90% des cas [4-7]; la douleur peut être selon le siège de la lésion soit cervicalgies voir même névralgies cervico-brachiales; névralgies intercostales ou des lombosciatalgies et parfois même des rachialgies diffuses. Elles sont habituellement de type mécanique à début insidieux; peu intense exacerbées par l'effort et calmée par le repos et les antalgiques; elle est rarement de type inflammatoire. Cette douleur s'accompagne dans la majorité des cas d'un syndrome infectieux modéré avec fébricule, altération de l'état général et à un stade évolué elle s'accompagne de fatigabilité musculaire voir des troubles neurologiques à type de déficit moteur. Les troubles neurologiques diffèrent d'une série à l'autre; ainsi pour Maftah et al [5] 50% des patients avaient un déficit neurologique, et pour Loembe et al [7] 81% des malades étaient déficitaires. 

Des déformations rachidiennes exprimées cliniquement par un simple décalage d'une apophyse épineuse, ou totalement une gibbosité majeure peuvent être observées, elles étaient retrouvées chez 34.1% des patients de notre étude, ce qui rejoint la série de Maftah et al [5]. Dans la série de Ghadouane et al [6], plus de la moitié (68.96%) avaient des déformations rachidiennes dominées par la cyphose. L'association de la tuberculose vertébrale avec d'autre localisation extra-rachidienne est très fréquente, et doit être recherché systématiquement.

L'apport de l'imagerie constitue incontestablement l'un des piliers du diagnostic du mal de Pott; elle permet de préciser à la radiographie standard le nombre de foyer atteint et les vertèbres intéressées; de montrer l'existence ou non d'un abcès cliniquement muet; d'éliminer une autre cause à l'origine de la symptomatologie; de rechercher d'autres lésions tuberculeuses (pulmonaire, ostéoarticulaire); et enfin de surveiller l'évolution des lésions [8-11]. Le pincement discal est le signe le plus précoce [1,10]. Les géodes représentent des lésions caractéristiques, mais non pathognomoniques. Elles peuvent être uniques ou multiples, arrondies ou ovalaires à contours plus ou moins flous, elles sont de taille variable. Ces géodes intéressent le plus souvent deux vertèbres adjacentes, et réalisent l'aspect classique de géodes en Â miroir Â de part et d'autre d'un disque pincé. Les séquestres osseux sont très évocateurs voir pathognomoniques de la nature tuberculeuse de la spondylodiscite, ils peuvent se présenter au sein des lésions géodiques ou au sein des abcès [12,13].

La tomodensitométrie (TDM), est une technique plus sensible que la radiographie standard dans le diagnostic de la spondylodiscite. Au stade de début, le disque intervertébral est le siège d'une hyperdensité évocatrice de lésion infectieuse. La destruction des plateaux est difficile à évaluer sur les coupes axiales, les reconstructions frontales ou sagittales sont en revanche très utiles pour rechercher des érosions et des géodes sous chondrales. La TDM permet également une bonne étude des parties molles paravertébrales, par la recherche des abcès. 

L'imagerie par résonance magnétique (IRM) dans le domaine de l'infection osseuse et plus particulièrement disco-vertébrales est devenue l'examen de référence après les radiographies standards. L'aspect habituel dans le mal de pott se traduit en séquence T1, par un hyposignal intéressant le disque et le corps vertébral, ce signal devient hyperintense en T2. L'injection de Gadolinium montre un rehaussement hétérogène du signal discosomatique ce qui permet de limiter les géodes et de rechercher l'atteinte des parties molles. L'IRM permet également la précocité du diagnostic, un bilan d'extension loco-régionale, de mettre en évidence des abcès intra et extracanalaire qui ont un très grand intérêt à la fois diagnostic et pronostique [14], et le diagnostic différentiel avec les autres spondylodiscites infectieuses et les lésions néoplasiques. 

L'intradermoréaction (IDR) constitue un élément de présomption diagnostic de tuberculose [15]; son pourcentage de positivité dans notre série s'élève à 85.36% ; ce chiffre est proche de celui de Loembe et al (86.36%). l'Anatomopathologie permet le diagnostic de certitude, en montrant le granulome épithélial et gigontocellulaire avec de la nécrose caséeuse [16,17]. On fait l'analyse anatomopathologique à une sensibilité de 72% [18]. En pratique, le diagnostic de certitude de tuberculose vertébrale est difficile à porter, et le plus souvent retenu sur des arguments radio-cliniques et conduit souvent à privilégier les épreuves thérapeutiques [19]. C'est pour cela que dans les pays à forte prévalence tuberculeuse on se contente d'un diagnostic de présomption pour démarrer les antibacillaires. 

La prise en charge thérapeutique du mal de pott reste encore controversée entre les différentes écoles. L'attitude vis-à-vis du foyer vertébral continue à être partagée entre le traitement médical exclusif et le traitement médico-chirurgical. La question qui se pose toujours quand faut-il opérer?, quel abord faut-il réaliser? Les antibacillaires peuvent guérir la tuberculose vertébrale à condition que l'on soit sûr du diagnostic et en absence de compression neurologique. Selon Debeyre et al [20] les antibacillaires doivent être administré à dose maximale d'emblée et sous forme d'une polychimiothérapie associant au moins trois antibacillaires; pour éviter toute résistance du BK, de façon continue et prolongée. La durée du traitement varie entre 6 et 18 mois.

Le principe général du traitement chirurgical reste le même, avec un abord antérieur large du foyer tuberculeux, une excision complète des lésions, une évacuation des abcès et élimination des séquestres osseux et discaux assurant une décompression du canal médullaire, puis le comblement de la perte de substance résultante par un greffon cortico-spongieux. Pour Andrey et al [21], en Afrique, l'intervention chirurgicale est habituelle, vu l'importance des lésions au stade du diagnostic; c'est la même conception qu'on retrouve chez les chirurgiens de l'extrême orient qui opèrent tous les spondylodiscites tuberculeuses. Dans notre étude, nous avons opérées 71.5% des malades, alors que chez Maftah et al ce chiffre était de 61.8%. Dans la littérature mondiale, Sefarino et Compt [22] et Fustec et al [23] ont opéré 41% de leurs malades, Luis et al [24] ont opéré 92% de leurs patients. De manière générale et comme dans notre série la chirurgie est indiquée dans la tuberculose vertébrale en cas de lésions très destructives avec instabilité, déformations rachidiennes importantes ou troubles neurologiques [25]. Au niveau de l'étage dorsal l'abord antérieur est difficile et consiste à une thoracotomie droite ou gauche selon la prédominance des lésions, trans ou rétropleurale selon l'expérience du chirurgien. La voie postéro latérale dans le mal de pott dorsal est adoptée par certains auteurs [7], elle consiste en la résection d'une ou deux apophyses transverses à leur base, et des arcs costaux correspondants à leur quart postérieure; on décolle la plèvre vers l'avant et en dedans pour découvrir assez largement la face antéro-latérale des corps vertébraux; cette méthode expose à des multiples risques surtout les brèches ostéoméningées [6]. Le mal de pott de la charnière dorsolombaire pose un problème thérapeutique du fait de la complexité anatomique de cette région. Selon Zlitini et Kassab [26], la laminectomie est une intervention à rejeter car elle ne permet qu'une décompression transitoire et peut aggraver une cyphose préexistante d'où l'importance de la voie antérieure consistant à une thoraco-phréno-lombotomie permettant un accès à l'ensemble de la charnière dorsolombaire; dans notre série, elle était réalisée chez 7.3% des patients; chez Ghadouane et al dans 6.89% des cas. 

Le mal de Pott lombaire d'accès relativement facile par voie antérieure (lombotomie rétro péritonéale), a en général un pronostic meilleur vu la rareté de l'atteinte neurologique et des déformations [6]; son diagnostic est le plus souvent posé au stade d'abcès de psoas. L'immobilisation plâtrée reste un complément thérapeutique classique chez tous les auteurs. La durée varie selon la localisation rachidienne. La rééducation est indispensable, son but est de compensée l'atrophie musculaire qui résulte de l'immobilisation, de prévenir les escarres, les attitudes vicieuses et de rééduquer les viscères, la musculature et la mobilité chez les paraplégiques. L'évolution clinique favorable sous traitement médical est la règle avec disparition de la fièvre, les douleurs rachidiennes s'estompent progressivement pour disparaître en quelques semaines. Sur le plan neurologique, le geste chirurgical de décompression par voie antérieure associé aux antibacillaires met le patient dans des meilleures conditions, pour une bonne récupération du déficit. Ceci était prouvé dans notre étude chez tous les malades opérés par voie antérieure. L'évolution radiologique des lésions tuberculeuses disco-somatiques traitées se fait en général vers La constitution d'un bloc osseux solide obtenue en moins d'un an. La guérison radiologique peut être obtenue avec persistance des images de destruction osseuse [26]. Certains éléments peuvent conditionner le pronostic dans le mal de Pott [27,28] comme la durée d'évolution de la maladie, l'état général du malade, l'état osseux et le statut neurologique avant la prise en charge et l'âge du malade ainsi que le traitement chirurgical antérieur ou postérieur. 

## Conclusion

A travers cette étude, nous constatons le mal de Pott touche souvent l'adulte jeune, issu d'un milieu défavorisé. Les deux sexes sont touchés de façon égale. Malheureusement plusieurs malades sont encore vus à un stade avancé de la maladie, avec des troubles neurologiques et des déformations rachidiennes très importantes. L'imagerie moderne (TDM et l'IRM) permet d'orienter le diagnostic, de donner le bilan morphologique et de planifier la stratégie thérapeutique. Le diagnostic de certitude exige la confirmation histologique, mais il peut être retenu sur des éléments cliniques et paracliniques de présomption. Dans les formes simples, un traitement médical seul bien conduit peut guérir le mal de Pott sans séquelle, alors que dans les formes compliquées, l'association d'un traitement médical à une chirurgie précoce surtout par voie antérieure met le patient dans les meilleures conditions pour une récupération neurologique et stabilisation rachidienne rapide. 

## Conflits d'intérêt

Les auteurs ne déclarent aucun conflit d'intérêts.

## Contribution des auteurs

Tous les auteurs ont également contribué à ce travail et ont lu et approuvé la version finale du manuscrit.
